# Electroencephalography (EEG) for neurological prognostication after cardiac arrest and targeted temperature management; rationale and study design

**DOI:** 10.1186/s12883-014-0159-2

**Published:** 2014-08-16

**Authors:** Erik Westhall, Ingmar Rosén, Andrea O Rossetti, Anne-Fleur van Rootselaar, Troels Wesenberg Kjaer, Janneke Horn, Susann Ullén, Hans Friberg, Niklas Nielsen, Tobias Cronberg

**Affiliations:** Department of Clinical Sciences, Division of Clinical Neurophysiology, Lund University, Lund, Sweden; Department of Neurology, CHUV and University of Lausanne, Lausanne, Switzerland; Department of Neurology/Clinical Neurophysiology, Academic Medical Center, University of Amsterdam, Amsterdam, The Netherlands; Department of Clinical Neurophysiology, Rigshospitalet University Hospital, Copenhagen, Denmark; Department of Intensive Care Medicine, Academic Medical Center, University of Amsterdam, Amsterdam, The Netherlands; R&D Centre Skane, Skane University Hospital, Lund, Sweden; Department of Clinical Sciences, Division of Intensive and Perioperative Care, Lund University, Lund, Sweden; Department of Anaesthesia and Intensive Care, Intensive Care Unit, Helsingborg Hospital, Helsingborg, Sweden; Department of Clinical Sciences, Division of Neurology, Lund University, Lund, Sweden

**Keywords:** EEG, Prognosis, Interrater variability, Brain injury, Out-of-hospital cardiac arrest, Hypothermia, Resuscitation

## Abstract

**Background:**

Electroencephalography (EEG) is widely used to assess neurological prognosis in patients who are comatose after cardiac arrest, but its value is limited by varying definitions of pathological patterns and by inter-rater variability. The American Clinical Neurophysiology Society (ACNS) has recently proposed a standardized EEG-terminology for critical care to address these limitations.

In the Target Temperature Management (TTM) trial, a large international trial on temperature management after cardiac arrest, EEG-examinations were part of the prospective study design. The main objective of this study is to evaluate EEG-data from the TTM-trial and to identify malignant EEG-patterns reliably predicting a poor neurological outcome.

**Methods/Design:**

In the TTM-trial, 399 post cardiac arrest patients who remained comatose after rewarming underwent a routine EEG. The presence of clinical seizures, use of sedatives and antiepileptic drugs during the EEG-registration were prospectively documented.

After the end of the trial, the EEGs were retrieved to form a central EEG-database.

The EEG-data will be analysed using the ACNS EEG terminology. We designed an electronic case record form (eCRF). Four EEG-specialists from different countries, blinded to patient outcome, will independently classify the EEGs and report through the eCRF. We will describe the prognostic values of pre-specified EEG patterns to predict poor as well as good outcome. We hypothesise three patterns to always be associated with a poor outcome (suppressed background without discharges, suppressed background with continuous periodic discharges and burst-suppression). Inter- and intra-rater variability and whether sedation or level of temperature affects the prognostic values will also be analyzed.

**Discussion:**

A well-defined terminology for interpreting post cardiac arrest EEGs is critical for the use of EEG as a prognostic tool.

The results of this study may help to validate the ACNS terminology for assessing post cardiac arrest EEGs and identify patterns that could reliably predict outcome.

**Trial registration:**

The TTM-trial is registered at ClinicalTrials.gov (NCT01020916).

## Background

Out of hospital cardiac arrest is a common cause of sudden death. It has a yearly incidence of 40–54 per 100 000 inhabitants [1,2], and an overall survival rate of 3%-10% [3-5]. Comatose survivors are admitted to an intensive care unit (ICU) and approximately half will die during hospital stay. While extensive hypoxic-ischemic brain injury is the main cause of death, severe neurological disability is uncommon among survivors [6].

For patients who remain in coma when sedative drugs have been cleared after rewarming, a neurological examination is the foundation for decisions whether or not to continue intensive care. Prediction of outcome is based on repeated clinical neurological examinations, most often combined with other methods of prognostication [7,8]. Accurate prediction requires test methods with a very high specificity, i.e. a very low false positive rate, for a poor outcome. A recent survey showed that, next to the clinical neurological examination, electroencephalography (EEG) is the most commonly used tool to assess prognosis after cardiac arrest (Friberg et al. Unpublished observation).

EEG is also important for detecting subclinical seizure activity in the sedated and sometimes paralyzed post cardiac arrest patient. Electrographic seizures and postanoxic status epilepticus are associated with a poor prognosis [9-15], but the electrographic definitions vary and there are several reports describing survivors with good neurological outcome [10,12,16,17]. Currently, there is no high-level evidence regarding whether treatment of electrographic seizures or even status epilepticus alters outcome after cardiac arrest. Guidelines on post-arrest care recommend an EEG to rule out seizure activity and for prognostication [18,19].

However, EEG has some important limitations when used in this setting. Firstly, it may be affected by sedation, which is routinely used during temperature management. A decreased body temperature prolongs the metabolism of sedative drugs and it may be difficult to know whether residual sedation still affects the patient or not [20].

Secondly, grading systems and definitions of malignant and benign EEG-patterns vary between studies, which complicate comparisons and meta-analysis. Most grading systems were developed decades ago, before the era of target temperature management [21-25]. In most recent studies, only a limited number of EEG-features were evaluated and it is reasonable to assume that the interpreting EEG-specialists could have been biased by other EEG features. Thus, a standardized terminology evaluating all important features would be preferable to assess which individual features of the EEG are the most predictive.

Thirdly, EEG-specialists usually are confident regarding their own interpretations, but considerable inter-rater variability has been described [26-28]. We are not aware of any previous studies on the prognostic value of post cardiac arrest EEG including an evaluation of inter- and intra-rater variabilities. Furthermore, most previous studies were performed at single centres by experienced researchers and the generalizability of the results is questionable.

The American Clinical Neurophysiology Society (ACNS) recently proposed a revised version of a standardized critical care EEG terminology [29], to characterize both rhythmic and periodic patterns as well as background activity. The intentions were to standardize terminology of the EEG patterns seen in encephalopathic patients to allow collaborative, multicentre studies and maximize inter-rater reliability.

The Target Temperature Management trial (TTM-trial) randomized 950 comatose cardiac arrest patients to a targeted body temperature of 33°C and 36°C post-arrest and found no significant differences in mortality or neurological function between the two groups [30].

In the TTM-trial, a routine EEG was performed in patients who were still comatose 12 hours after rewarming. Overall, 399 EEGs were performed. This protocol describes the rationale and methods of the EEG-analysis of the TTM-trial.

### Aims

We will investigate the prognostic value, as well as intra- and inter-rater variability of specific EEG patterns in a routine EEG performed after rewarming, according to the ACNS standardized EEG terminology.

Our main objective is to identify highly malignant patterns that predict a poor neurological outcome with a zero false positive ratio (FPR) and acceptable inter-rater variability.

### Hypotheses

1. For patients who have suffered a cardiac arrest and remain in coma after rewarming, the following EEG features (defined according to ACNS terminology) are:
  - always associated with a poor outcome (“highly malignant EEG” with a FPR = 0%):
    Suppressed background (<10 μV) without Discharges.
    Suppressed background (<10 μV) with Continuous Periodic Discharges.
    Burst-suppression background (with or without Discharges) with suppression periods (<10 μV) constituting >50% of the recording.
  - nearly always associated with a poor outcome (“malignant EEG” with a FPR < 5%):
    Periodic or rhythmic patterns:
    Abundant Periodic Discharges (>50% of recording).
    Abundant Rhythmic SW (=polyspike-/spike-/sharp-and-wave) (>50% of recording).
    Unequivocal electrographic seizure (at least one).
    Background EEG:
    Discontinuous background with suppression periods (<10 μV) constituting >10% of the recording.
    Low voltage background (most activity <20 μV).
    Reversed anterior-posterior gradient.
    Reactivity:
    Absence of background reactivity or only stimulus-induced discharges (SIRPIDs) for both sound and pain stimuli.
  - associated with a good outcome in the majority of cases (“benign EEG”):
    Absence of all malignant features stated above.
2. The prognostic ability of a malignant EEG improves if more than one of the following malignant features (stated in hypothesis 1b) are present in the same EEG:
  Malignant periodic or rhythmic pattern.
  Malignant background EEG.
  Unreactive background.
3. The prognostic ability of a benign EEG improves if combined with presence of background reactivity for either sound or pain stimuli.
4. The prevalence and prognostic ability of highly malignant, malignant and benign EEGs after rewarming are similar between patients treated with 33°C or 36°C.
5. The prognostic ability of highly malignant and malignant patterns is similar between patients that were affected by sedation and those that were not. Influence of sedation is defined as ongoing sedation during the EEG-registration or if the treating physician considered the patient affected by residual sedation on the day of EEG-registration.
6. There is an acceptable inter- and intra-rater variability (Kappa >0,6) for grouping EEG patterns into the highly malignant, malignant and benign categories stated above and for all the individual features of the ACNS EEG terminology.

## Methods/Design

### Ethical approval and trial registration

The TTM-trial protocol included a routine EEG and was approved by the ethics committees in all participating countries and institutions. The TTM-trial is registered at ClinicalTrials.gov (NCT01020916) and the trial protocol has been published elsewhere [31].

### Recording of EEGs in the TTM-trial

The recording of EEG-data in the TTM-trial was pre-specified [31] and approved by the TTM steering group. The TTM-trial recruited patients from November 2010 to January 2013. A routine EEG was performed according to the trial protocol in patients that remained unconscious 12 hours after rewarming.

Routine EEGs were performed at all 36 sites (intensive care units in Europe and Australia) in the trial. Sites were given recommendations on how to perform the EEG-registrations including the use of ≥16 EEG-channels and testing of background reactivity for both sound and pain stimuli. The result (EEG-report) of the routine EEG was used at each centre according to local routine. The presence of seizures, sedatives, antiepileptic medication and the level of consciousness were prospectively documented in the TTM-database.

A physician blinded for target temperature level performed a neurological evaluation 72 hours after rewarming and issued a recommendation for the continuation or withdrawal of life-sustaining therapy. In the TTM-trial, persisting coma (Glasgow Coma Scale-Motor 1–2) 72 hours after rewarming in combination with a treatment refractory postanoxic status epilepticus defined by EEG was one of the findings that allowed discontinuation of active intensive care [31].

### Study population

Adult patients resuscitated after out-of-hospital cardiac arrest of presumed cardiac cause that were still unconscious on admission to the hospital. The patients were randomized to target temperature management at 33°C or 36°C. Sedation was mandated in both groups until after rewarming, 36 hours after randomisation.

### Study design for the EEG analysis

For the EEG analysis, four EEG-specialists (Sweden: *EW*, Denmark: *TWK*, The Netherlands: *AFvR,* and Switzerland: *AOR*) were invited together with members of the steering group, with special interest in neurophysiology, to form the EEG group of the TTM-trial (May 2013).

### EEG database

On behalf of the TTM-trial steering group, a central EEG database was created at the department of Clinical Neurophysiology, Skane University Hospital, Lund, Sweden. All 36 site investigators were contacted and the local EEG laboratories were encouraged to provide anonymised routine EEGs in electronic format (EDF+).

All notations in the EEGs were translated to English. Notations that were important for the interpretation of the EEGs were kept, for instance notations concerning artefacts, electrode adjustments, movements of the patient, clinical seizure descriptions and if significant changes in level of sedation occurred during the registration. Information that could obviously bias the EEG-specialists for instance notations about the level of consciousness, including voluntary movements and clinical reaction to stimuli, and results of cranial nerve examination were removed. All data on gender and age were also removed.

### Inclusion criteria

A total of 399 patients had a routine EEG according to the TTM-database, all will be considered for inclusion in this study.

### Exclusion criteria

1. EEGs performed before reaching normothermic body temperature.
2. EEGs not yet received from the sites before start of analysing phase three (autumn 2014).
3. EEGs that do not meet the following quality criteria:
  electronic format compatible with the EEG-specialists EEG-readers.
  ≥8 electrodes + reference electrode + ground electrode.
  registration time of ≥10 minutes.

### EEG analysis

The EEGs will be analysed using the American Clinical Neurophysiology Society (ACNS) standardized critical care EEG terminology [29]. The four EEG-specialists will independently perform the EEG-analysis, after studying the terminology and ACNS web based training module (www.acns.org).

### First phase (pilot study)

Twenty EEGs, chosen to cover important aspects of the ACNS EEG terminology, were analysed during the autumn 2013 by the four EEG-specialists. The purpose was to evaluate inter-rater variability for all categories of the ACNS EEG terminology. For this pilot study paper case report forms were used to report the EEG-findings. There was moderate agreement on the presence and type of periodic and rhythmic patterns [32].

### Second phase

A web based eCRF was created (Experlytics AB) for the EEG analysis. The eCRF contains a limited number of categories from the ACNS EEG terminology. The categories were chosen after evaluating inter-rater variability in the pilot study and clinical relevance (literature study, see Table 1). Ten categories were excluded (typical duration, longest duration, phases, relative amplitude, polarity, onset, symmetry, breach effect, posterior dominant rhythm, variability) due to low kappa values (<0,4). In addition, these categories were considered less relevant for evaluation of post cardiac arrest EEGs.

**Table 1:** **Previous publications describing malignant EEG patterns are presented**

**Described malignant EEG patterns in previous publications**	**Features of the ACNS EEG terminology included in the hypotheses of the present study**	**Number of previous publications:**					
		**Treated with TTM**			**Not treated with TTM**		
		**FPR = 0**	**FPR < 5**	**FPR > 5**	**FPR = 0**	**FPR < 5**	**FPR > 5**
		**PPV = 100**	**PPV > 95**	**PPV < 95**	**PPV = 100**	**PPV > 95**	**PPV < 95**
“Burst-suppr”	Burst-suppr (suppr >50%)	3 [10,33,34]	1 [35]	0	5 [36-40]	1 [41]	0
“Discontinous background with suppr >1 sec”	Discontinuous background (suppr >10%)	0	0	0	3 [22,39,42]	0	0
“Low voltage background (<20 μV)”	Low voltage background (most activity <20 μV)	1 [33]	1 [10]	0	8 [22,36-42]	0	0
“Alfa coma pattern”	Reversed anterior-posterior gradient	1 [33]	0	0	5 [22,36,37,43,44]	1 [45]	3 [39,46,47]
“PDs on a flat background”	Continuous PDs *and* Voltage = suppressed (<10 μV)	0	0	0	2 [22,36]	0	0
“Continuous Epileptiform Activity or Electrographic Status Epilepticus (broad definition)”	Abundant PDs *or* Abundant Polyspike-/Spike-/Sharp-and-Waves	3 [14,33,48]	1 [10]	0	1 [39]	0	1 [38]
“Electrographic seizure activity”	Unequivocal seizures	1 [33]	0	0	0	0	0
“Unreactive background”	Reactivity absent *or* SIRPIDs only	3 [33,49,50]	0	0	1 [39]	0	1 [51]

Malignant EEG patterns after cardiac arrest described in previously published studies [10,14,22,33-51] are presented according to their predicting values (PPV) and false positive ratio (FPR) and whether target temperature management (TTM) were applied or not. We have listed the features (or combinations of features) of the American Clinical Neurophysiology Society’s critical care EEG terminology, that we consider correspond to the previously published malignant patterns, which are chosen to be tested in the hypotheses of this study. They were chosen since at least one previous study reported FPR < 5% or PPV > 95% for corresponding EEG patterns. The patterns were thus considered to have a potential to become markers for poor prognosis. For some studies the described malignant patterns were part of an EEG grading system and therefore the prevalence of these patterns could not be decided. PDs = Periodic Discharges, SIRPIDs = Stimulus-induced rhythmic, periodic, or ictal discharges.

In the second phase, 103 EEGs will be analysed independently and in a randomized order by the four EEG-specialists during 2014. These EEGs represent all EEGs from 8 selected sites, which were chosen as they routinely performed testing of reactivity. The 20 EEGs analysed in the first phase will also be included in the 103 EEGs of the second phase for the purpose of evaluating intra-rater variability. When the EEG analysis of the second phase is completed, we will analyse inter- and intra-rater variability (hypothesis 6).

### Third phase

We estimate that an additional 200 EEGs will be included in the third phase. In some of these EEGs, testing of background reactivity will not have been performed.

### Outcome measures

All patients were followed-up until 180 days after cardiac arrest. The outcome measures were neurological function and mortality. The hypotheses will be correlated to outcome using the cerebral performance category scale (CPC) [52]. Good outcome is defined as CPC 1–2 at 180 days after cardiac arrest or a best achieved CPC of 1–2 at any time. Poor outcome is defined as best achieved CPC of 3–5 during the first 180 days after cardiac arrest.

## Statistical analysis

### Descriptive statistics

Prevalence will be presented for all individual features in hypotheses 1a-c as well as grouping the features into “highly malignant EEG”, “malignant EEG”, “benign EEG” as well as subgroups of “malignant EEG”: “malignant periodic or rhythmic patterns”, “malignant background EEG” and “unreactive EEG”. The prevalence will be presented as range of the findings reported by the four interpreters as well as a “majority decision”. The “majority decision” for the findings of an individual EEG means that if two or more interpreters have reported a feature, this EEG will be judged to have this feature. If two interpreters have reported one feature and the two others interpreters have reported another feature, the most malignant feature will be reported if the “malignancy” of the features can be graded (i.e. “suppressed background” is more malignant than “burst-suppression background”). If the features cannot be graded (i.e. “low voltage background” versus “reversed anterior-posterior gradient”) both features will be presented.

### Prognostic ability

The evaluation of the prognostic ability of EEG will be presented by specificity, sensitivity and false positive rate (FPR), defined as 1-specificity. Similarly as the prevalence, these values will be given both as a range of findings reported by the four interpreters as well as a “majority decision”. Prognostic measures will be compared between groups using a generalized linear mixed model including values from all four interpreters.

### Inter-rater variability

Inter-rater variability (Kappa and percent agreement) will be presented for all individual features of the ACNS EEG terminology as well as grouping the features according to hypotheses 1a-c (“highly malignant EEG”; “malignant EEG”; “benign EEG”, “malignant periodic or rhythmic patterns”; “malignant background EEG”; “unreactive EEG”).

### Sample size

Following the primary hypothesis (1a) we assume a FPR of 0 and the study is planned to include approximately 400 patients among whom 125 had a good neurological outcome. Based on these assumptions a 95%-confidence interval (using Wilson’s method) of FPR will be 0-3%.

## Discussion

Earlier studies of hypothermia-treated patients found that approximately one third of patients are still comatose 3 days after rewarming [53] and we found the proportion of patients that had an EEG in the TTM-trial (n = 399, 42%) to be close to the expected. Thereby, the 399 EEGs are likely to compose a representative sample of EEGs performed for the purpose of prognostication.

EEG has been used for decades for prognostication after cardiac arrest, but its accuracy as a prognostic method is limited by lack of consensus on definitions of malignant patterns [54-56] and by inter-rater variability [26-28].

The recently revised standardized critical care EEG terminology by ACNS [29] also includes characterization of the background activity, which was lacking in the earlier version [57]. Thereby, it has become possible to use the terminology to investigate the prognostic significance of different EEG patterns in comatose cardiac arrest patients.

Our main objective is to identify highly malignant patterns with an acceptable level of inter-rater variability that can reliably be used to aid in prognostication and in decisions on whether or not to withdraw life sustaining treatment or continue active care. The terminology was designed to maximize inter-rater reliability, which will also be tested. Previously described malignant EEG patterns were recently reviewed [54-56] and we used the evidence on malignant patterns from these three reviews to design the hypotheses of our study (Table 1).

The much used American Academy of Neurology’s (AAN) practice parameters did not consider patients treated with temperature management and listed three EEG-patterns as highly malignant; generalized suppression, burst-suppression with generalized epileptiform activity, and generalized periodic complexes on a flat background; these correspond to the highly malignant patterns in the hypotheses of our study. Since the definition of burst suppression in the ACNS terminology is rather conservative (suppression periods constituting more than 50% of the recording), we hypothesised that burst-suppression is a highly malignant pattern per se, even without discharges.

It is likely that our restriction of highly malignant patterns to those with a false positive ratio (FPR) of zero will lead to a low sensitivity for detecting poor outcome. In order to increase the sensitivity of EEG to detect poor outcome, we will also test other features of the ACNS terminology that we consider potentially malignant (an expected FPR <5%). We therefore selected patterns with a FPR <5% or a PPV >95% in at least one of the previously published studies (Table 1). The corresponding categories of the ACNS terminology were considered to have a potential to become markers of a poor prognosis and were therefore termed “malignant” in the hypotheses of this study.

We used the CPC categories 3–5 to define poor neurological outcome as was done in the TTM-trial and in accordance with the majority of EEG-studies in recent years [55,56].

EEG is affected by sedation [58]. The TTM-trial registered information about ongoing sedation during the routine EEG and whether the treating physician judged that sedation was still affecting the level of consciousness of the patient. Since the routine EEG was performed after rewarming, sedation is likely to have been tapered for most patients. If some EEG-patterns will prove predictive of outcome despite residual sedation, this would be clinically relevant information. We therefore chose not to exclude EEGs judged to be affected by sedation from our main hypotheses, but to analyse the effect of sedation (considering it a dichotomous variable) separately (hypothesis 5).

Postanoxic status epilepticus was previously described as a malignant pattern [9-13]. Based on our own experience and previously published studies using routine or continuous EEG monitoring, we expect the majority of status epilepticus to have started and thus be detected at the time point of the routine EEG in the TTM-trial [10]. Since a treatment refractory status epilepticus in combination with persisting coma 72 hours after rewarming was one of the findings that allowed discontinuation of active intensive care, the validity of the prognostic value may have been affected by early withdrawal of care, a phenomenon known as self-fulfilling prophecy.

Background reactivity has been described to have important prognostic implications [33,49,50,59] and all sites were recommended to perform reactivity testing for sound and pain stimuli. However, this was not performed in all sites and in addition some EEG software systems could not export notations about stimulations. Therefore, we will only be able to evaluate reactivity in approximately 150 EEGs.

It is of paramount importance to establish a well-defined and comprehensible terminology for interpretation of EEG, especially when EEG is used as a prognostic tool and as a support for decisions on whether active care should be continued or withdrawn. The ACNS EEG terminology is standardized and available for all EEG-specialists. The results of our study should help establish this terminology for assessment of prognosis in comatose post cardiac arrest patients.

## Abbreviations

AAN: American academy of neurology
ACNS: American clinical neurophysiology society
CA: Cardiac arrest
CPC: Cerebral performance category scale
eCRF: Electronic case report form
EDF+: European data format
EEG: Electroencephalography
FPR: False positive ratio
GCS: Glasgow coma scale
NPV: Negative predictive value
PPV: Positive predictive value
SIRPIDs: Stimulus-induced rhythmic, periodic, or ictal discharges
SW: Polyspike-/spike-/sharp-and-wave
TTM: Target temperature management
